# *In vitro* assessment of probiotic potential of selected bacteria isolated from pig faeces with potential application of odour reduction

**DOI:** 10.1080/23144599.2021.1936962

**Published:** 2021-07-02

**Authors:** M Jahangir Alam, Mahfuzul Islam, Che-Ok Jeon, Ki-Choon Lee, Seon-Ho Kim, Chul-Ju Yang, M Enayet Kabir, Sang-Suk Lee

**Affiliations:** a Department of Animal Science and Technology, Sunchon National University, Suncheon, Republic of Korea; b Faculty of Animal Science and Veterinary Medicine, Sher-e-Bangla Agricultural University, Dhaka, Bangladesh; c Department of Life Science & Research Center for Biomolecules and Biosystems, Chung-Ang University, Seoul, Republic of Korea; d Department of Animal Sciences, The Ohio State University, Columbus, OH, USA

**Keywords:** Bacterial effects, *in vitro* fermentation, pig odour reduction, polymerase chain reaction

## Abstract

To evaluate the odour reduction potential of four different bacterial species such as *Enterococcus faecium, Enterococcus faecalis, Acetobacter tropicalis*, and *Bacillus subtilis* subsp. *subtilis* that were isolated from fresh faeces of pigs and identified based on16S rDNA gene sequence analyses. Faecal slurry in anaerobic salt medium with 1% soluble starch (which was served as control group) and the addition of four different isolated bacterial cultures (1.0 × 10^7^CFU/mL), designated as M1, M2, M3, and M4, respectively, were incubated anaerobically for 12 and 24 h. Total gas production was increased with the incubation period (*p < 0.05*). M1 and M4 had decreased pattern (*p < 0.05*) of ammonia and hydrogen sulphide gas from 12 to 24 h. The lowest total volatile fatty acids (*p* < 0.05), highest lactate, and moderate butyrate concentration was observed in the M1 group at 24 h of incubation. Likewise, M1 group had the lowest total biogenic amine, histamine, ethylamine, putrescine, methylamine, and cadaverine compared to the other groups (*p* < 0.05) at 24 h of incubation. Overall results suggest that *E. faecium* can be used as a potent odour reducer in pigs production.

## 1. Introduction

The odorous compounds generated from pig rearing are hazardous to farmers and pigs [1–4]. To alleviate the odour problem, strict environment regulations are continually being strengthened throughout the world [5,6]. Digestive function in the large intestine primarily involves the microbial breakdown of carbohydrates and proteins to short-chain fatty acids (SCFA), amines, and different gases under anaerobic conditions, which are the principal precursors of odour production [7]. The malodorous compounds produced mostly in the large intestine, through the degradation of several substrates by the microbial actions [8]. The dietary carbohydrates remained undigested in the small intestine reached in to the hindgut where they contributed to produce odorous compounds through microbial degradation in the pig [9]. Various carbohydrate concentrations (0.25–5.0 g/100 mL) were evaluated on caecal fermentation which was measured by the changes in pH, and maximal effects were elicited with 1.0 g/100 mL of starch [10]. Accordingly, 1.0 g of starch (as carbohydrates) was used to initiate carbohydrate overload in this experiment.

Many options are available to reduce odour released from pig production. The biofiltration methods are proven efficient to reduce odour emission in pig building by many researchers [11–13]. However, they can be difficult to operate and more expensive than other odour reduction strategies in terms of construction cost [14]. Also, chemical methods such as oxidizing agents, was applied to reduce obnoxious odours from pig house; however, those have relatively short periods of effectiveness [15] and can be potentially toxic to farmers and pigs if applied excessively [16]. Few laboratories have tested the ability of certain feed supplements to reduce or eliminate odorous emissions *in vivo* [17]. Recently, a pilot study was conducted to reduce odour through slurry application method [18]. Besides, these treatments are done with the faeces after excretion into the environment and these methods simultaneously could not have met standards of efficiency, economics and safety. On the other hand, effectiveness of biological additives for odour abatement is comparatively weaker than biofiltration and the chemical method [19], but they have the advantages of low cost, easy treatment, and non-toxicity [14]. For instance, the ammonia and hydrogen sulphide emissions were reduced by the inoculation of *Lactobacillus casei* strain as a potential candidate under *in vitro* conditions for the management of animal waste [20]. Hong and Lee [21] also stated that some microorganisms showed their ability to decompose the odorous substances. Therefore, identification of suitable microbial-based digestive additives that can enhance beneficial microbial communities is essential. Indeed, manipulation of the gut microbial community by probiotics can reduce the adverse environmental effect by swine production. Therefore, the present study was undertaken to select different types of isolated microbes from pig faeces and evaluate the odour reduction potentiality of these bacteria by *in vitro* fermentation system. The data obtained from this *in vitro* study will be helpful in selecting bacterial strain (s) that could possibly be a promising candidate for pig waste management.

## 2. Materials and methods

### 2.1. Ethical statement

The study was conducted at the Sunchon National University (SCNU) animal farm and the ruminant nutrition and anaerobe laboratory, Department of Animal Science and Technology, SCNU, Jeonnam, South Korea. All experiment and animals used in this study were approved by the SCNU Institutional Animal Care and Use Committee (IACUC) (approval no. SCNU IACUC-2019-12).

### 2.2. Isolation and identification of bacterial strain

#### 2.2.1. Sample collection and isolation of bacteria

The isolation and identification of bacteria were performed following the protocol of Cotta et al. [22] with minor modification. Briefly, for isolation of bacterial strain, the faecal samples were directly collected from the rectum of pig. The samples were then mixed with a salt medium for the anaerobic jar fermentation. Samples from the jar fermentation were diluted and cultured on agar media (tryptic soy and MRS) (Merck, Germany) by spread plate method and incubated at 37°C for 24 h for *Bacillus*, and 48 h for lactobacilli. Selected bacterial colonies were sub-cultured to made pure culture. The pure bacterial colonies were then grown in their selective broth (tryptic soy for *Bacillus* and MRS for lactobacilli) and preserved the stock cultures in a cryovial (with 15% glycerol) at -20°C.

#### 2.2.2. Polymerase chain reaction (PCR)

The colony PCR were performed to confirm the isolated bacteria. The DNA of purified single colony was extracted by 5% chelex (Bio-Rad, California, USA). DNA products of the suspension were used for 16S rDNA amplification via PCR which is called colony-PCR. The universal primers, 27 F (5′-AGAGTTTGATCMTGGCTCAG-3′) and 1492 R (5′-GGYTACCTTGTTACGACTT3′), were employed to amplify the bacterial 16S rDNA [23]. PCR was performed using the *Taq* DNA Polymerase Kits (Promega, WI, USA) following the manufactural instructions. A Gene Amp PCR System of PTC-100 thermal cycler (MJ Research, Inc., Watertown, MA, USA) was used to amplify the samples. PCR condition was as follows: 94°C for 3 min then, 94°C for 1 min, 50°C for 1 min, and 72°C for 1 min for 30 continuous cycles and 72°C for 7 min as a final extension. Visualization of the amplicons was performed by a Gel Logic 200 imaging system (Eastman KODAK Company, Rochester, NY) after electrophoresis with ethidium bromide to confirm the sizes (1.5 kb).

#### 2.2.3. DNA sequencing and phylogenetic tree construction

The amplified PCR products were purified by using QIAquick PCR Purification Kits (Qiagen, Valencia, CA, USA). The purified DNA were sent to the Macrogen (Seoul, Korea) for sequencing and the analyzed sequence fragments were assembled by using the SeqMan programme (DNA Star, Lasergene software, Madison, WI). The BLAST programme (http://www.ncbi.nlm.nih.gov/BLAST/) was used to compare the resultant 16S rDNA gene sequences with the available 16S rDNA gene sequences in the GenBank of NCBI, and EzTaxon was used to determine an approximate phylogeny. The gene sequences were aligned with those of closely related species using CLUSTAL W version 1.6 [24]. Phylogenetic trees were constructed via the neighbour-joining (NJ) method with pair-wise gap removal. Distance matrices were calculated using the Kimura two-parameter model [25] of the NJ method in the PHYLIP package, and bootstrap analysis was conducted by running the data 1000 times to evaluate the stability of the phylogenetic tree. Only bootstrap values in excess of 50% are shown on the internal nodes [26].

### 2.3. In vitro fermentation

**Figure 1.**
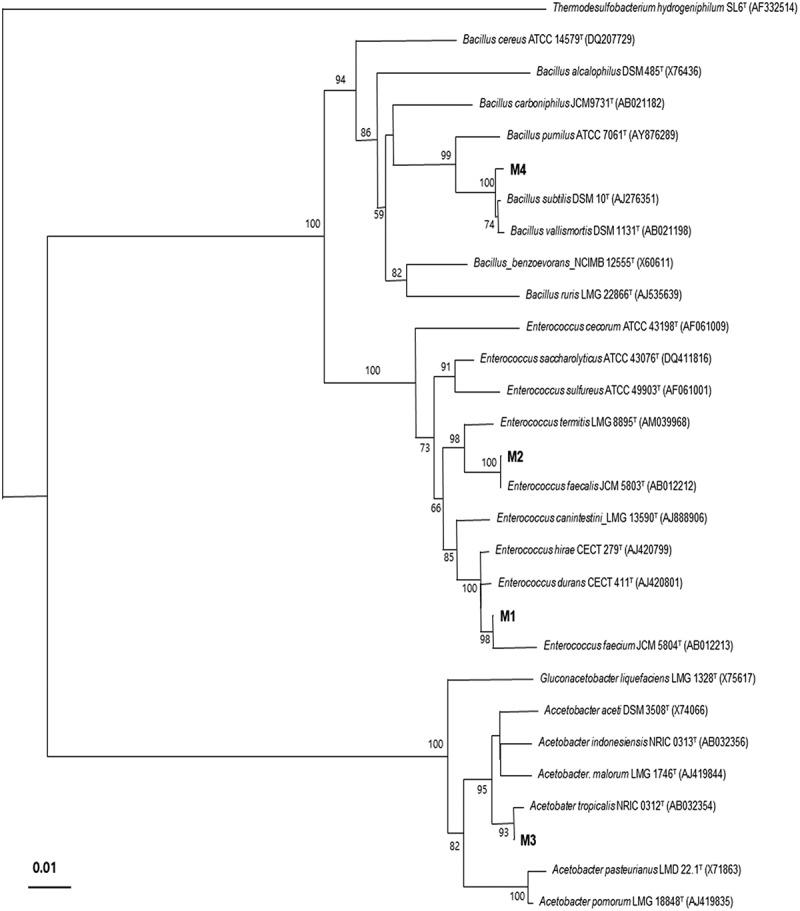
Phylogenetic tree of the isolated bacterial strains from faeces of pigs based on 16S rDNA gene sequences produced by the Kimura two- parameter correction models and constructed using the neighbour-joining method. The tree was bootstrap re-sampled 1,000 times. Only bootstrap values in excess of 50% are shown on the internal nodes. *Thermodesulfobacterium hydrogeniphilum* SL6^T^ was used as an out-group. Bar, 0.01 substitutions per nucleotide position; M1, *Enterococcus faecium*; M2, *Enterococcus faecalis*; M3, *Acetobacter tropicalis*; M4, *Bacillus subtilis* subsp. *subtilis.*

Four isolated bacterial strains designated as M1, M2, M3, and to M4 (Figure 1 and Table 1) were used to evaluate their odour reduction potential through *in vitro* experiment. The identified selected microbes were grown in the selected broth to make as the microbial cultures. For *in vitro* fermentation, fresh faeces were directly collected from the recta of the pigs and transferred in to a thermo flask maintained temperature at 38°C under vacuum, which was generally done by 15 minutes to prevent extraneous contamination. Collected faeces were added into the salt medium at 10% (w/v) under a constant flow of CO_2_ [9]. The above-mentioned salt medium was prepared earlier according to the methods previously described by Jensen et al. [27] and Wang et al. [9]. This medium was pH adjusted (6.0 ± 0.3) and autoclaved. The prepared suspension was homogenized and filtered through a 4-folded sterile cheese cloth. Sterile serum bottles (160 mL) were then inoculated with 100 mL prepared faecal slurry and 1 g soluble starch (Yakuri, Kyoto, Japan), and bacterial cultures (1.0 × 10^7^CFU/mL) under anaerobic conditions. The serum bottles were subsequently placed in a shaking incubator (HB 201SF, Hanbaek Scientific Co., Korea) at 50 rpm and 38°C for 12 and 24 h. The amount of total gas (TG), pH, the concentration of ammonia-nitrogen (NH_3_–N; both gas and liquid phase), hydrogen sulphide (H_2_S), volatile fatty acid (VFA) and biogenic amines (BA) were observed at each incubation time of *in vitro* fermentation.

**Table 1.** List of identified bacteria isolated from faeces of pigs with their nearest relative and identity

Sample No.^a^	Nearest relative	Identity
*Enterococcus faecium* (M1)	*Enterococcus faecium* JCM 5804	99.0%
*Enterococcus faecalis* (M2)	*Enterococcus faecalis* JCM 5803	99.8%
*Acetobacter tropicalis* (M3)	*Acetobacter tropicalis* NRIC0312	99.8%
*Bacillus subtilis* subsp. *subtilis* (M4)	*Bacillus subtilis* subsp. *subtilis* NCIMB3610	99.7%

^a^M1 to M4 represent microbes compared as different treatments.

### 2.4. Odour compounds analysis of in vitro fermentation

At each incubation period, TG production was measured by a press and sensor machine (Laurel Electronics, Inc., Costa Mesa, CA). The concentration of ammonia (NH_3_) and H_2_S gas was measured by using a VRAE, Multi Gas Monitor (PGM-7840, RAE Systems Inc., Sunnyvale, CA) prior to sampling for other analysis. The pH was measured immediate after uncapping of the bottles and the samples were stored at −20°C until further analysis. The NH_3_–N (liquid phase) concentration was measured using spectrophotometer (Libra S22; Biochrom Ltd., CB40FJ, England), according to the protocol of Chaney and Marbach [28]. VFA concentrations (acetate, propionate and butyrate), and lactate were measured by using a HPLC (Agilent Technologies 1200 series, Germany) according to the protocol developed by Tabaru et al. [29] and Han et al. [30]. BA separation was performed by using a Waters liquid chromatography (Waters Ltd., Massachusetts, USA). BA compounds were identified and quantified using standard curves constructed from the pure compounds (histamine, methylamine, ethylamine, tyramine, putrescine, and cadaverine) [31]. Samples for BA were centrifuged at 16,609 × g for 5 minutes at 4°C and filtered through 0.2 µm Millipore filters. Filtrates were hydrolyzed and evaporated using an Eyela SB-1000 (Tokyo Rikakikai Co. Ltd., Japan).

### 2.5. Statistical analysis

All data were subjected to one-way ANOVA procedures under a completely randomized design, using the general linear models procedures (SAS Inst. Inc., Cary, NC) [32]. The effect of the microbe on total gas, pH, NH_3_–N (both gas and liquid phase), H_2_S, VFA, lactate, and BA concentrations were compared and examined significant differences among means of treatment and control groups using Duncan’s multiple range (comparison) tests. Significant differences among treatments was declared by *P* < 0.05 for the variables measured.

## 3. Results

There were four bacterial species, such as *Enterococcus faecium* (M1), *Enterococcus faecalis* (M2), *Acetobacter tropicalis* (M3), and *Bacillus subtilis* subsp. *subtilis* (M4), which were isolated from pig faeces which were confirmed by sequencing and phylogeny (Table 1 and Figure 1). The result of this experiment revealed that the total gas production increased with the elapsing incubation period (Table 2). Total gas production at both stages differed significantly with different microbes (*p* < 0.05). The pH value at 12 h was high in M1 and M3 treatments and the lowest was recorded in control and M2 treatment, while at 24 h of incubation, it was high in control and lowered in the other treatments (*p* < 0.05) (Table 2). The concentrations of NH_3_ gas was rapidly decreased from 12 to 24 h, and the concentration was not detected in the M1 while the detection was low in the M3 group at 24 h incubation (*p* < 0.05). The lowest concentration of NH_3_ in the gas phase was observed in M1 at both stages. On the other hand, NH_3_–N concentrations in the liquid phase increased in M1 and M4, while decreased in M2 and M3 at 12 h with notable reduction in M1 at 24 h (*p* < 0.05) (Table 2). H_2_S concentrations was rapidly decreased in the M1, M3 and M4 treatment groups, but were slightly increased in the control and M2 groups from 12 to 24 h (*p* < 0.05) (Table 2). H_2_S was decreased towards zero in the M1, M3 and M4 groups at 24 h of microbial fermentation.

**Table 2.** Effect of identified bacteria on the changes of *in vitro* pH, total gas, ammonia-nitrogen, and hydrogen sulphide production at different incubation times

Parameters	Period (h)	Treatments					SEM^1)^
		Con^2)^	M1^3)^	M2^4)^	M3^5)^	M4^6)^	
pH values	12	5.52 ^c^	5.75^a^	5.52 ^c^	5.73^a^	5.63^b^	0.11
	24	5.55^a^	5.40^b^	5.51^a^	5.44^b^	5.42^b^	0.11
Total gas production, ml/g^7)^	12	31.67^b^	28.67 ^c^	27.33 ^c^	28.0 ^c^	43.33^a^	0.74
	24	37.0 ^c^	51.67^a^	53.67^a^	47.67^b^	47.67^b^	0.96
NH_3_–N (gas) content, mg/L	12	200.0^a^	20.67^d^	142.0^b^	21.67^d^	96.67 ^c^	1.82
	24	97.67^b^	ND^9)^	121.33^a^	0.67^d^	14.67 ^c^	0.86
NH_3_–N (liquid phase) content, mg/dl^8)^	12	5.04^b^	6.58^a^	3.0 ^c^	2.89 ^c^	6.31^a^	0.28
	24	8.47^a^	2.40^d^	6.63^b^	8.43^a^	5.04 ^c^	0.23
H_2_S gas content, mg/L	12	285.67^b^	293.0^a^	290.33^a^	292.0^a^	292.0^a^	1.14
	24	290.67^a^	ND	292.33^a^	ND	ND	1.05

Values presented as Mean from three replication in each; M1–M4 contained 1.0 × 10^7^CFU/mL of microbial culture in salt medium, faecal slurry and 1% soluble starch. ^a,b,c,d^ Means within rows with different superscripts differ (*P* < 0.05);

^1)^Standard error of the mean.

^2)^Control contained salt medium, faecal slurry, and 1% soluble starch.

^3)^*Enterococcus faecium*.

^4)^*Enterococcus faecalis*.

^5)^*Acetobacter tropicalis*.

^6)^*Bacillus subtilis* subsp. subtilis.

^7)^Total gas production in mL/g starch (DM basis) was used as substrate in the fermenta using the equation of *y* = 0.023*x*+0.055 (*R*^2^ = 0.996).

^8)^NH_3_–N (liquid phase) was calculated by the equation of *y* = 0.0004*x* + 0.0002 and the standard, *R*^2^ = 0.9998.

^9)^Not detected.

Acetate concentration was higher in control, but lower in microbes added treatments at 24 h of incubation (*p* < 0.05) (Table 3). Propionate concentration was comparatively lower in M1, higher in M3, control, and M2, and medium in the M4 groups at 24 h. Butyrate concentration was medium in control, M1, and M4, but a slight high in M2, and M3 groups (*p* < 0.05) at 24 h incubation. At 24 h, the lowest total VFA concentration was recorded in the M1 group followed by M4, M2, M3, and control (*p* < 0.05). The higher to lower order of lactate concentration was detected as M1 > control > M4 > M3 > M2 at 24 h (*p* < 0.05).

**Table 3.** Effect of identified bacteria on the changes of *in vitro* volatile fatty acids and lactate concentration at different incubation times

Parameters	Period (h)	Treatments					SEM^1)^
		Con^2)^	M1^3)^	M2^4)^	M3^5)^	M4^6)^	
Acetate (mmol/L)	12	10.18^a^	2.26 ^d^	3.70 ^c^	ND^7)^	5.11^b^	0.08
	24	18.12^a^	1.34 ^b^	ND	ND	1.94^b^	0.06
Propionate (mmol/L)	12	28.12^b^	48.66^a^	24.63 ^c^	21.06^d^	27.62^b^	0.37
	24	30.34^b^	9.68^d^	29.62^b^	41.21^a^	25.47 ^c^	0.40
Butyrate (mmol/L)	12	5.95^e^	10.33 ^c^	39.24^a^	9.33^d^	20.61^b^	0.17
	24	7.84^e^	9.56 ^c^	11.03^b^	14.06^a^	8.90^d^	0.17
Total VFA (mmol/L)	12	44.25 ^c^	61.25^a^	67.56^a^	30.39^d^	53.35^b^	0.18
	24	56.30^a^	20.58 ^c^	40.65^b^	55.27^a^	36.31^b^	0.53
Lactate (mmol/L)	12	17.04^bc^	17.16^b^	12.52^d^	28.53^a^	16.36 ^c^	0.19
	24	18.13^ab^	18.50^a^	14.10^d^	17.23 ^c^	17.55^bc^	0.16

VFA, volatile fatty acids.

Values presented as Mean from three replication in each;

M1–M4 contained 1.0 × 10^7^CFU/mL of microbial culture in salt medium, faecal slurry and 1% soluble starch ^a,b,c,d^ Means within rows with different superscripts differ (*P* < 0.05).

^1)^Standard error of the mean;.

^2)^Control contained salt medium, faecal slurry, and 1% soluble starch.

^3)^*Enterococcus faecium*.

^4)^*Enterococcus faecalis*.

^5)^*Acetobacter tropicalis*.

^6)^*Bacillus subtilis* subsp. subtilis.

^7)^Not detected.

In the case of histamine, the lowest concentration (*p* < 0.05) was detected in the M1 group followed by the M4, M3, M2 and control groups at 24 h of *in vitro* fermentation (Table 4). M1 group also had lowest methylamine and ethylamine levels compared to other groups at 24 h (Table 4). Putrescine levels were ranged from lower to higher in M1, control, M2, M4 and M3, respectively, at 24 h of fermentation. At 24 h of incubation, cadaverine concentration was also lowest in the M1 group, followed by control, M4, M2, and M3, respectively. At 24 h, total BA concentrations accounted from higher to lower as control > M2 > M3 > M4 > M1 (*p* < 0.05). Tyr was observed in control; however, not detected in the treatments. Total BA concentration was decreased from 12 h to 24 h of fermentation in all groups, except the control and M2 groups where it was increased, and the lowest concentration was observed in the M1 group.

**Table 4.** Effect of identified bacteria on the changes of *in vitro* biogenic amines production at different incubation times

Parameters	Period (h)	Treatments					SEM^1)^
		Con^2)^	M1^3)^	M2^4)^	M3^5)^	M4^6)^	
His (mg/L)	12	197.17^a^	101.67 ^c^	73.02^e^	92.66^d^	153.33^b^	2.92
	24	301.27^a^	16.70 ^c^	295.43^a^	23.16^b^	20.00^b^	2.50
Meth (mg/L)	12	1.55^b^	1.57^b^	0.29 ^c^	0.20 ^c^	1.76^a^	0.10
	24	1.58^ab^	1.01 ^c^	1.26^b^	1.72^a^	1.22^b^	0.06
Ethy (mg/L)	12	5.20 ^b^	1.60^d^	1.80 ^d^	7.48^a^	2.18 ^c^	0.15
	24	3.12^b^	0.33^e^	2.31 ^c^	8.02^a^	0.91^d^	0.15
Tyr (mg/L)	12	0.20^a^	ND^7)^	ND	ND	ND	0.002
	24	0.33^a^	ND	ND	ND	ND	0.01
Putre (mg/L)	12	1.93 ^c^	0.48^d^	3.54^b^	11.11^a^	4.74^b^	0.29
	24	1.78 ^c^	1.71 ^c^	2.86^bc^	12.12^a^	3.26^b^	0.28
Cad (mg/L)	12	4.89^b^	0.91^d^	3.56 ^c^	10.78^a^	3.40 ^c^	0.22
	24	0.77 ^c^	0.76 ^c^	1.30^b^	11.71^a^	0.87 ^c^	0.16
TBA (mg/L)	12	210.94^a^	106.23^d^	82.21^e^	122.23 ^c^	165.41^b^	2.96
	24	308.84^a^	20.51 ^c^	303.16^a^	56.73^b^	26.26 ^c^	2.40

His, histamine; Meth, methylamine; Ethy, ethylamine; Tyr, tyramine; Putre, putrescine; Cad, cadaverine; TBA, total biogenic amine.

Values presented as Mean from three replication in each;

M1–M4 contained 1.0 × 10^7^CFU/mL of microbial culture in salt medium, faecal slurry and 1% soluble starch. ^a,b,c,d^ Means within rows with different superscripts differ (*P* < 0.05).

^1)^Standard error of the mean.

^2)^Control contained salt medium, faecal slurry and 1% soluble starch.

^3)^*Enterococcus faecium*.

^4)^*Enterococcus faecalis*.

^5)^*Acetobacter tropicalis*.

^6)^*Bacillus subtilis* subsp. subtilis.

^7)^Not detected.

## 4. Discussion

In this study, four different bacterial species such as *Enterococcus faecium, Enterococcus faecalis, Acetobacter tropicalis*, and *Bacillus subtilis* subsp. *subtilis* were isolated from fresh faeces of pig. Metiner et al. [33] also isolated *Enterococcus faecium* and *Enterococcus faecalis* from pig faeces. Earlier studies reported that some bacteria had the potential to reduce malodour from swine farm [21,34,35]. Therefore, we hypothesized that the bacteria used in this study can be reduced odorous compound from pig production.

It was observed that the pH tended to decrease with an elapsing fermentation period in most cases, except in the control, which did not include microbes. Lactic acid is an intermediary product of carbohydrate fermentation and accumulates only when VFA production is inhibited in an acidic milieu with a pH of <5.5 [36,37]. This correlation between pH and lactate was consistent with our findings and previous studies. Risley et al. [38] and van Kempen [39] demonstrated that lowering the pH of urine and subsequent slurry is beneficial for reducing odour and ammonia emissions. Bailey et al. [10] showed that anaerobic incubation with either corn starch or insulin induced significant time-dependent reductions in pH from 0 to 24 h. In our experiment, pH was reduced more profoundly in the microbial groups than in the control, possibly as the result of increased acid production (lactate or others); this result is also consistent with the above-mentioned findings.

Basic principle of gas production is that the *in vitro* feeds fermentation by microorganisms is accompanied by the gas production [40,41]. TG production observed in the present study was in agreement with Groot et al. [42] and Patra et al. [43], who reported that TG production, and odorous compounds increased with the elapsing of incubation period of microbial fermentation. However, an increased odour is not reflected by the increased gas production rather it depends on the types of odorous compounds produced. Therefore, we attempted to evaluate different microbes after analysing some of these gaseous compounds. Our findings demonstrated that, in certain cases, NH_3_ concentrations were reduced after *in vitro* microbial fermentation. In an experiment conducted by Naidu et al. [20], they selected a *Lactobacillus casei* strain capable of reducing ammonia and hydrogen sulphide emissions under *in vitro* conditions. Thus, we selected different types of isolated microbes, some of which reduced H_2_S, and NH_3_–N (both gas and liquid phase). M1 and M4 principally reduced NH_3_–N concentrations in both the gas and liquid phases. Nahm [44] reported that fibre-degrading bacteria utilize ammonia as a substrate for microbial protein synthesis, and are subsequently excreted in the faeces. NH_3_ concentrations were not detected in the gas phases by the M1 group at 24 h, possibly as the result of rapid microbial utilization of NH_3_ as a substrate for microbial protein synthesis or conversion to others as described above. Although the results of the present experiment were not obtained with microbes similar to those used in previous studies, we were able to confirm the desired reduction of odour with selective *Enterococcus* (M1) and *Bacillus* (M4) microbial fermentation. Fakhoury et al. [45] and Suarez et al. [46] identified S-compounds as important for malodour production and they implicated that H_2_S had the highest correlation with malodour. It was reported earlier by Gibson et al. [47] that the S-reducing bacteria can utilized hydrogen in the terminal stages of fermentation which can prevent excess accumulation of gas in the colonic lumen. In the U.S.A., *Lactobacillus* strains (Pat. No. 4,345,032 and 4,879,238) were disclosed, which showed a reduction trend of odorous substances like sulphides, ammonia or VFAs [48,49]. In the present experiment, we selected different isolated bacteria from pig faeces, some of which significantly reduced H_2_S levels (*P < 0.05*). Most notably, H_2_S was not detected in the M1, M3, and M4 groups (*Enterococcus, Acetobacter* and *Bacillus*) at 24 h of microbial fermentation. In our experiment, even though we used different microbes than those used by previous researchers [20,35,48–50] for reducing sulphide production in *in vitro* fermentation which is consistent with prior research trends.

The VFAs account for most of (around 90%) the malodorous substances in faeces [51,52]. The degradation compounds from fermentable carbohydrates typically are SCFAs such as acetic acid, propionic acid, butyric acid, valeric acid, etc [53,54]. Potter et al. [55] explained that dietary starch, when present in excess, may be fermented by caecal and colonic Gram-positive bacteria to generate lactic acid. Engberg et al. [56] determined that dietary organic acids reduced the numbers of observed microbial forms in both the ileum and caecum, but the lactic acid bacteria (LAB) were affected less profoundly. LAB are usually considered to provide health benefits to the host, maintain pH and inhibiting the growth of potentially pathogenic Gram-negative flora, e.g. *E. coli* and *Salmonella*. A novel *Bacillus* strain was isolated from soil by Yumoto et al. [57] that was able to deodorize SCFA; however, we isolated different groups of bacteria from fresh faeces of pigs. Moreover, previous studies have shown that addition of carbohydrates can modulate the microflora in the digestive system of pigs [58,59] to alter VFA patterns in the gastrointestinal tract and reduce odorous compounds from swine manure [60]. Our principal objective was to reduce the odour emanating from the pig. The results showed that total VFA were reduced from 12 to 24 h using some of the microbes during fermentation. M1 and M4 comparatively reduced more total VFA at 24 h of incubation. Butyrate production was noted to be relatively moderate with these 2 treatments as compared to the others. Lactic acid production, which is beneficial to the health of animals as well as energy production, was comparatively better with M1 and M4 treatments, which shows evidence relating to the reduction of pH level. Although energy production may be reduced to some degree by the lowering of total VFA, it will facilitate odour reduction. Moreover, it is the last stage of fermentation and maximum odour compounds excreted through faeces rather than effective energy supply that causes odour production. Possible reason according to Liao and Bundy [61] and Barrington [62] to decrease the odour compounds, microbial additives contain bacteria or bacterial related enzymes that eliminate odours and suppress gaseous pollutants by their biochemical digestive processes.

Biogenic amines are produced primarily by the decarboxylation of certain amino acids by microbial action. Gram-positive bacteria such as streptococci and lactobacilli, as well as Gram-negative species, possess the ability to generate amines from amino acids [63]. Amines have a disagreeable odour, can cause nausea, and have a toxic effect [64]. Bailey et al. [65] identified microorganisms which are capable of reducing the amount or concentration of toxic and odorous compounds in faeces. Therefore, these microorganisms are able to reduce the generation of faeces odour and toxicity. An experiment was conducted by Bailey et al. [10] using the horse as a non-ruminant animal to observe BA production and reduction in the large intestine. They demonstrated that the fermentation of excess carbohydrates was associated with increased production of phenylethylamine, putrescine, cadaverine, etc. which may be reduced by using selected equine caecal microbiota. Furthermore, Bastos et al. [66] reported that direct-fed miocrobials (*Bacillus subtilis* and *Bacillus licheniformis*) inclusion reduced biogenic amines concentrations from faeces of dogs. The results of the present experiment demonstrated that some of the different selected microbes reduced BAs concentration with elapsing fermentation period. The M1 and M4 groups evidenced higher reductions of total BA, histamine, methylamine, ethylamine, putrescine and cadaverine than were observed with the other selected microbes at 24 h of fermentation. Present findings also confirmed that excess carbohydrates without selected bacterial fermentation stimulate increases in amine production (control).

## 5. Conclusions

In conclusion, this study addresses the use of probiotic microorganisms to reduce the generation of swine odour. Among the four isolates, *Enterococcus faecium* had the highest potential in terms of the reduction of odorous compounds. According to priority, *E. faecium* (preferred) and *Bacillus subtilis* subsp. *subtilis* can be used singly or multiply to generate probiotics that may efficiently reduce odorous compounds during fermentation in the large intestines of pigs. The limitation of the present study is that the experimental results were obtained only *in vitro* fermentation which will be overcome in the subsequent study through *in vivo* trial.

## Data Availability

The data presented in this study are available from the corresponding author upon reasonable request.
